# Causal relationship between the administration of high-dose corticosteroids and the appearance of maniform-type psychopathology

**DOI:** 10.1192/j.eurpsy.2023.1305

**Published:** 2023-07-19

**Authors:** M. O. Solis Correa, M. Valverde Barea, L. Soldado Rodriguez

**Affiliations:** Hospital Neurotraumatologico, Jaén, Spain

## Abstract

**Introduction:**

To present a clinical case that reflects the causal relationship between the administration of high-dose corticosteroids and the appearance of maniform-type psychopathology.

**Objectives:**

Descriptive study of a case report and literature review on the subject.

**Methods:**

32-year-old woman with alcohol abuse detected, added Antabus 250 mg / day to her treatment.

**Results:**

After 2 months of treatment, she was admitted to the Digestive Service due to acute hepatitis. After a liver biopsy and autoimmunity study was diagnosed as Autoimmune Hepatitis. Treatment with Antabus was withdrawn, and Prednisone 60 mg/day was prescribed. Seven days after starting treatment with corticosteroids, she presented maniform symptoms (psychomotor restlessness, expansive mood, dysphoria, megalomanic delusions, alteration of biological rhythms with decreased need for sleep and risk behaviors), and she was admitted in a psychiatric hospitalization unit. After considering various differential diagnoses she is diagnosed with Substance-Induced (corticosteroids) Mood Disorder with manic features. Psychiatry agrees with the Digestive Service to start treatment with Paliperidone and progressively lower the dose of corticosteroids until suspending it and prescribe an immunosuppressant. Finally, the maniform symptoms that led to admission remitted completely and control and outpatient treatment were continued.

**Conclusions:**

Its important to always keep in mind the great risk of the appearance of psychiatric disorders that treatment with high doses of corticosteroids entails, especially in susceptible patients or with a psychiatric history or genetic susceptibility. It is necessary to know the possible appearance of these neuropsychiatric adverse effects in order to prevent them, and if it appear, to assess, if possible, the suspension or reduction of corticosteroid treatment.

**Disclosure of Interest:**

None Declared

